# Mitochondrial depolarization promotes calcium alternans: Mechanistic insights from a ventricular myocyte model

**DOI:** 10.1371/journal.pcbi.1008624

**Published:** 2021-01-25

**Authors:** Vikas Pandey, Lai-Hua Xie, Zhilin Qu, Zhen Song

**Affiliations:** 1 Department of Medicine, David Geffen School of Medicine, University of California, Los Angeles, California, United States of America; 2 Department of Cell Biology and Molecular Medicine, Rutgers, New Jersey Medical School, Newark, New Jersey, United States of America; 3 Department of Computational Medicine, David Geffen School of Medicine, University of California, Los Angeles, California, United States of America; 4 Peng Cheng Laboratory, Shenzhen, Guangdong, China; University of Michigan, UNITED STATES

## Abstract

Mitochondria are vital organelles inside the cell and contribute to intracellular calcium (Ca^2+^) dynamics directly and indirectly via calcium exchange, ATP generation, and production of reactive oxygen species (ROS). Arrhythmogenic Ca^2+^ alternans in cardiac myocytes has been observed in experiments under abnormal mitochondrial depolarization. However, complex signaling pathways and Ca^2+^ cycling between mitochondria and cytosol make it difficult in experiments to reveal the underlying mechanisms of Ca^2+^ alternans under abnormal mitochondrial depolarization. In this study, we use a newly developed spatiotemporal ventricular myocyte computer model that integrates mitochondrial Ca^2+^ cycling and complex signaling pathways to investigate the mechanisms of Ca^2+^ alternans during mitochondrial depolarization. We find that elevation of ROS in response to mitochondrial depolarization plays a critical role in promoting Ca^2+^ alternans. Further examination reveals that the redox effect of ROS on ryanodine receptors and sarco/endoplasmic reticulum Ca^2+^-ATPase synergistically promote alternans. Upregulation of mitochondrial Ca^2+^ uniporter promotes Ca^2+^ alternans via Ca^2+^-dependent mitochondrial permeability transition pore opening. Due to their relatively slow kinetics, oxidized Ca^2+^/calmodulin-dependent protein kinase II activation and ATP do not play significant roles acutely in the genesis of Ca^2+^ alternans after mitochondrial depolarization, but their roles can be significant in the long term, mainly through their effects on sarco/endoplasmic reticulum Ca^2+^-ATPase activity. In conclusion, mitochondrial depolarization promotes Ca^2+^ alternans acutely via the redox effect of ROS and chronically by ATP reduction. It suppresses Ca^2+^ alternans chronically through oxidized Ca^2+^/calmodulin-dependent protein kinase II activation.

## Author summary

Mitochondrial dysfunction has been implicated in life-threatening cardiac arrhythmias. Experimentalists have shown that mitochondrial depolarization could cause period-doubling behavior in cytosolic Ca^2+^ transient (i.e., Ca^2+^ alternans). However, the underlying mechanisms behind these experimental observations remain unclear. Here, we focus on revealing the underlying mechanisms of cellular Ca^2+^ alternans in the context of mitochondrial depolarization. Using our newly developed action potential model that incorporates a 3D network of Ca^2+^ release unit with a 3D network of mitochondria, we dissect out individual roles of mitochondrial Ca^2+^ and complex signaling pathways (e.g., reactive oxygen species, oxidized Ca^2+^/calmodulin-dependent protein kinase II activation, and ATP) in the genesis of cellular Ca^2+^ alternans. The key finding is that mitochondrial depolarization promotes Ca^2+^ alternans acutely via the redox effect of ROS and chronically by ATP reduction, and oxidized Ca^2+^/calmodulin-dependent protein kinase II activation may suppress Ca^2+^ alternans chronically. We believe that this study reveals insightful information for understanding the mechanisms of the genesis of Ca^2+^ alternans in the context of mitochondrial depolarization and provides valuable simulation results to guide future experiments.

## Introduction

Calcium (Ca^2+^) is a critical regulator of excitation-contraction coupling in cardiac myocytes [1]. Ca^2+^ enters a cardiac myocyte mostly through the L-type Ca^2+^ channels (LCCs), which open in response to membrane depolarization. The resulting rise in the intracellular Ca^2+^ concentration activates ryanodine receptors (RyRs) to release a large amount of Ca^2+^ from the sarcoplasmic reticulum (SR), an internal Ca^2+^ store in cardiac myocytes. RyRs are clustered on the membrane of SR, forming discrete Ca^2+^ release units (CRUs). The SR Ca^2+^ release causes a transient increase in cytosolic Ca^2+^, which binds to myofilament to cause contraction. Ca^2+^ in the cytosol is extruded outside the cell via Na^+^-Ca^2+^ exchangers (NCX) and transported back to the SR through the sarco/endoplasmic reticulum Ca^2+^-ATPase (SERCA) pump. The normal Ca^2+^ cycling is essential to maintain the normal function of excitation-contraction coupling due to the bi-directional coupling through nonlinear interactions of ion channels, transporters, and pumps. Disturbances in the complex regulations of these components of the cell can lead to various nonlinear dynamics that underlie life-threatening cardiac arrhythmias [2]. Ca^2+^ alternans is one of the most studied phenomena for cardiac arrhythmias. Ca^2+^ alternans can cause action potential duration (APD) alternans due to the bi-directional coupling of the membrane voltage and Ca^2+^ [2,3]. Ca^2+^ and APD alternans are known to occur in acute myocardial ischemia and other diseased conditions [4–9]. APD alternans gives rise to T-wave alternans, which is a risk marker for sudden cardiac death [10,11]. Besides SR, mitochondria are also internal Ca^2+^ stores, and Ca^2+^ enters into and moves out of the mitochondria during a cardiac cycle, directly affecting intracellular Ca^2+^ signaling [8,12–17]. Moreover, mitochondria produce ATP and reactive oxygen species (ROS), which affect Ca^2+^ dynamics via their effects on the Ca^2+^ cycling proteins. Recent studies have shown that mitochondrial Ca^2+^ cycling plays a key role in cardiac diseases [18–22]. The goal of this work is to investigate mitochondrial contributions to the genesis of Ca^2+^ alternans.

Experimental studies have shown that metabolic stress or impaired mitochondrial function promotes Ca^2+^ alternans [14,23–26]. However, the complex effects of mitochondria on Ca^2+^ cycling, energy metabolism, and signaling make it difficult to dissect out the underlying mechanisms in experiments. Ca^2+^ enters mitochondria via the mitochondrial Ca^2+^ uniporter (MCU) [27,28], and exits mitochondria via mitochondrial sodium-calcium exchanger (mNCX) [29] or the mitochondrial permeability transition pore (mPTP). The mPTP open probability is very low under normal conditions but can be high under pathophysiological conditions. In addition to the direct mitochondrial Ca^2+^ cycling, mitochondria are coupled with intracellular Ca^2+^ cycling in several other ways. ROS signaling and the redox regulation affect the opening of RyRs and the activity of the SERCA pump [30,31]. Mitochondria provide ATP to the SERCA pump and other sarcolemmal ion pumps, and a shortage of ATP may impair the function of these pumps [32,33]. Moreover, there is a positive feedback loop between mitochondrial ROS production and SR Ca^2+^ release, i.e., leaky RyRs trigger more Ca^2+^ release resulting in more Ca^2+^ sequestered in mitochondria, which can trigger the opening of mPTP, leading to mitochondria depolarizations [34,35]. Mitochondrial depolarization produces ROS, which regulates SR Ca^2+^ uptake and release. The oxidized Ca^2+^/calmodulin-dependent protein kinase II (CaMKII) activation enhances the sensitivity of the SERCA pump through phosphorylation of phospholamban [36]. Concurrently, CaMKII also affects the activation of LCCs [37].

Therefore, mitochondria play complex roles in intracellular Ca^2+^ cycling via different pathways. These pathways can either promote or suppress alternans depending on their properties [38,39], making it very difficult to experimentally dissect out the key pathways responsible for alternans. We recently developed a physiologically detailed ventricular myocyte model that incorporated mitochondrial Ca^2+^ cycling, mPTP, ROS production, and oxidized CaMKII signaling [40]. We showed that this model could exhibit Ca^2+^ alternans and spontaneous Ca^2+^ release mediated delayed afterdepolarizations (DADs) under mitochondrial depolarization. In the present study, we use computer simulations of this model to investigate the underlying mechanisms for the genesis of Ca^2+^ alternans in response to mitochondrial depolarization induced by mPTP opening. Taking advantage of computer simulations, we can differentiate the effects of direct mitochondrial Ca^2+^ cycling, the redox regulation of RyRs and SERCA by ROS, oxidized CaMKII signaling, and ATP reduction on Ca^2+^ alternans. We demonstrate that mitochondrial depolarization promotes Ca^2+^ alternans acutely via the redox effect of ROS and chronically by ATP reduction, and may suppress Ca^2+^ alternans chronically by oxidized signaling, mainly via their effects on SERCA activity.

## Results

### Ca^2+^ alternans caused by mitochondrial depolarization due to mPTP opening

**Fig 1:**
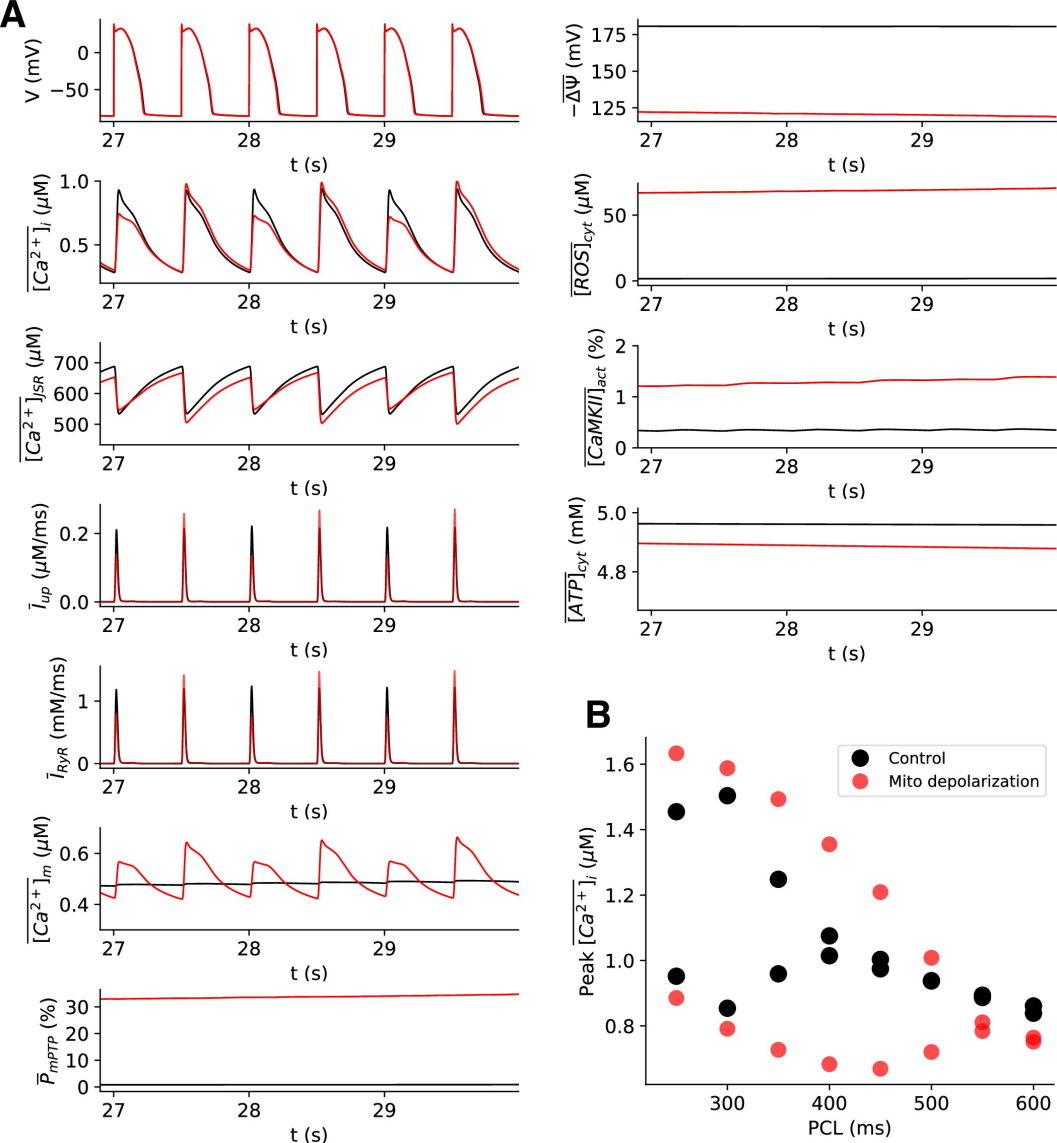
Mitochondrial depolarization promotes Ca^2+^ alternans. **A.** Time traces of membrane potential V, whole-cell averaged cytosolic Ca^2+^ concentration ${\bar{\left[Ca^{2+}\right]}}_{i}$, SR Ca^2+^concentration ${\bar{\left[Ca^{2+}\right]}}_{JSR}$, SERCA uptake flux, SR Ca^2+^ release flux via RyRs, mitochondrial free Ca^2+^ ${\bar{\left[Ca^{2+}\right]}}_{m}$, open probability of mPTP ${\bar{P}}_{mPTP}$, mitochondrial membrane potential $-\bar{\Delta \psi }$, cytosolic ROS concentration ${\bar{\left[ROS\right]}}_{cyt}$, cytosolic CaMKII activation ${\bar{\left[CaMKII\right]}}_{act}$, cytosolic ATP concentration ${\bar{\left[ATP\right]}}_{cyt}$ for normal control (*α*_*mPTP*_ = 1) in black and mitochondrial depolarization (*α*_*mPTP*_ = 60) conditions in red, respectively. PCL is 500 ms. Note that the horizontal bar above each variable means that the quantity is an averaged value over all the CRUs or mitochondria within the myocyte. **B.** Bifurcation diagrams of peak values of ${\bar{\left[Ca^{2+}\right]}}_{i}$ in the last two consecutive beats vs. PCL for control (black) and mitochondrial-depolarization (red) cases.

To show the effects of mPTP opening on Ca^2+^ alternans, we carried out simulations under the control condition (where the mPTP open probability is very low) and a high mPTP open probability condition (by increasing the transition rate from the closed state, C_1_, to the open state, O, to 60-fold of the control value, i.e., *α*_*mPTP*_ = 60) for PCL = 500 ms (Fig 1A). Under the control condition (black lines), no alternans occurred. Under the condition of high mPTP open probability (red lines), Ca^2+^ alternans occurred. And the Ca^2+^ alternans was abolished when all the mPTPs within the cell were commanded to be in the closed state, which also recovered Δ*ψ* (S1 Fig). These observations were consistent with our previous experimental results using either mPTP inhibitor or cyclophilin D knockout mouse model [41] in which mitochondrial depolarization was prevented. Note that the corresponding APD alternans appears small (see the enlarged one in S2A Fig), since the Ca^2+^ alternans amplitude is small (~0.2 *μ*M) at PCL = 500 ms. At a faster pacing rate (PCL = 300 ms), the APD alternans becomes more significant due to greater Ca^2+^ alternans amplitude (S2B Fig). Note that for this *α*_*mPTP*_ value, the open probability of mPTP was about 30%. Also, note that at t = 30 s CaMKII activity and cytosolic ATP are still changing slowly. When we ran the simulations for a much longer time (e.g., 1000 s), the CaMKII activity and the cytosolic ATP became ~74% and ~2 mM, respectively. Since these two variables change extremely slowly over time, we treated them as quasi-steady state variables for the half-minute long simulation. For the same reason, we investigated the acute effect of mitochondrial depolarization on the genesis of Ca^2+^alternans in this study by performing free-running simulations for 30 s. Fig 1B shows a bifurcation diagram plotting the peak values of cytosolic Ca^2+^ transient against the pacing cycle length (PCL) for both conditions. The high mPTP open probability condition changed the onset of Ca^2+^ alternans to a longer PCL, from 450 ms to 550 ms.

Since mPTP opening and mitochondrial depolarization can affect the intracellular Ca^2+^ dynamics directly or indirectly via changing the properties of Ca^2+^ handling proteins (e.g., RyRs and SERCA) or altering CaMKII and other signaling pathways [30,31], it becomes difficult to reveal the roles of each process experimentally. In the following sections, we take advantage of computer simulation to examine the individual roles of cytosolic ROS, mitochondrial Ca^2+^, CaMKII activation, and cytosolic ATP in the genesis of Ca^2+^ alternans.

### Effects of ROS on the genesis of Ca^2+^ alternans

**Fig 2:**
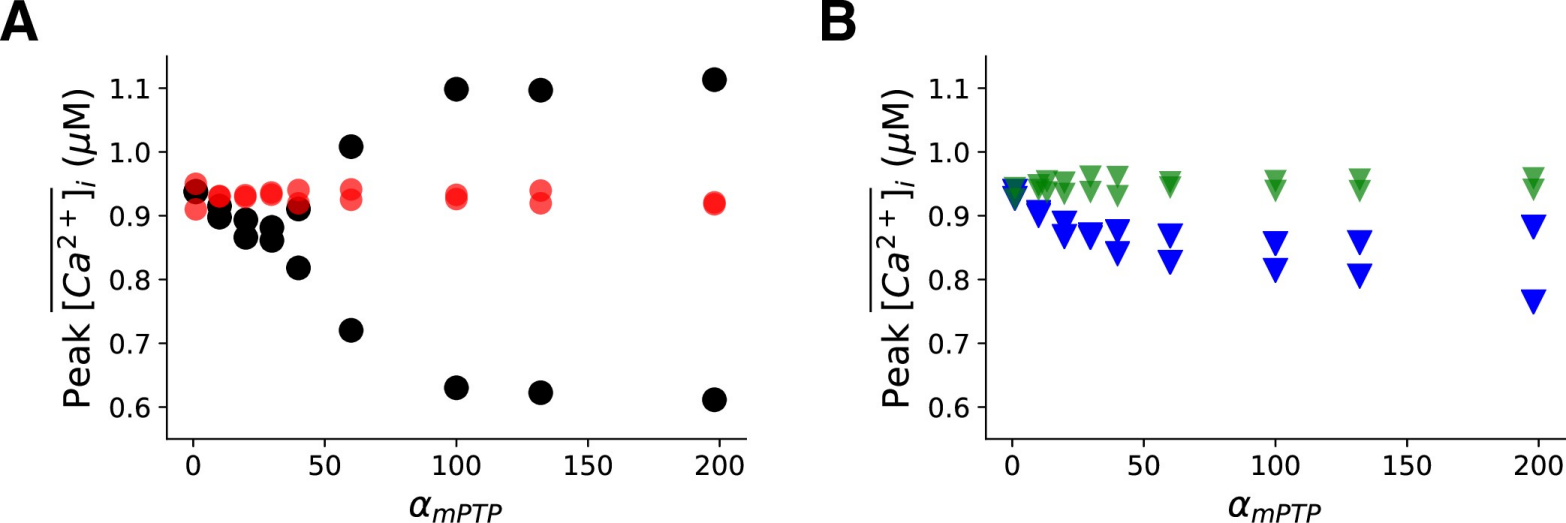
Effect of cytosolic ROS signaling on the genesis of Ca^2+^ alternans. **A**. Bifurcation diagram of peak values of Ca^2+^ transients vs. *α*_*mPTP*_ for free-running ROS (black) and fixed ROS = 0.1 *μ*M (red), respectively. **B.** Bifurcation diagram of peak values of Ca^2+^ transient vs. *α*_*mPTP*_ with free-running ROS, but the redox effect of ROS only exerted on RyRs (green) or SERCA (blue), respectively. The PCL is 500 ms. The total simulation time for each simulation is 30 sec.

To determine the effect of ROS on Ca^2+^ alternans, we carried out simulations in the conditions of the free-running ROS (i.e., the ROS dynamics obeys the differential equations) and a clamped ROS (i.e., the ROS was fixed to a constant) at PCL = 500 ms, shown in Fig 2A as bifurcation diagrams. Under the free-running case, increasing α_mPTP_ above certain threshold promotes alternans, but when ROS was clamped at a low level (0.1 μM), no alternans occurs. Therefore, our simulation results here suggest that Ca^2+^ alternans was predominantly mediated by the cytosolic ROS. Also, note that increasing α_mPTP_ increases the open probability of mPTP, and under the setting of the free-running ROS, we found that Ca^2+^ alternans became obvious when the open probability of mPTP is above ~20% (S3 Fig).

To further dissect out how ROS promotes Ca^2+^ alternans, we first investigate its redox effect on RyRs and SERCA separately. Fig 2B shows the bifurcation diagrams for the redox effect of ROS on RyRs only (green, by setting *f*_*up*,*ros*_ = 1 in Eq 5) or for the redox effect of ROS on SERCA only (blue, by setting Δ*k*_*ros*_ = 0 in Eq 2). We then performed the same simulations as in Fig 2A for these two cases (Fig 2B). When the redox effect on SERCA was removed, the amplitude of Ca^2+^ alternans was greatly reduced (Fig 2B, green). When the redox effect on RyRs was removed, the amplitude of Ca^2+^ alternans was also decreased (Fig 2B, blue) compared to the case of free-running ROS (Fig 2A, black). Note that the onsets of alternans for these two cases are about the same as in the case of free-running ROS (Fig 2A, black). These results suggest that the redox effect of ROS on RyRs and SERCA synergistically promotes Ca^2+^ alternans.

### Effects of mitochondrial Ca^2+^ on the genesis of Ca^2+^ alternans

**Fig 3:**
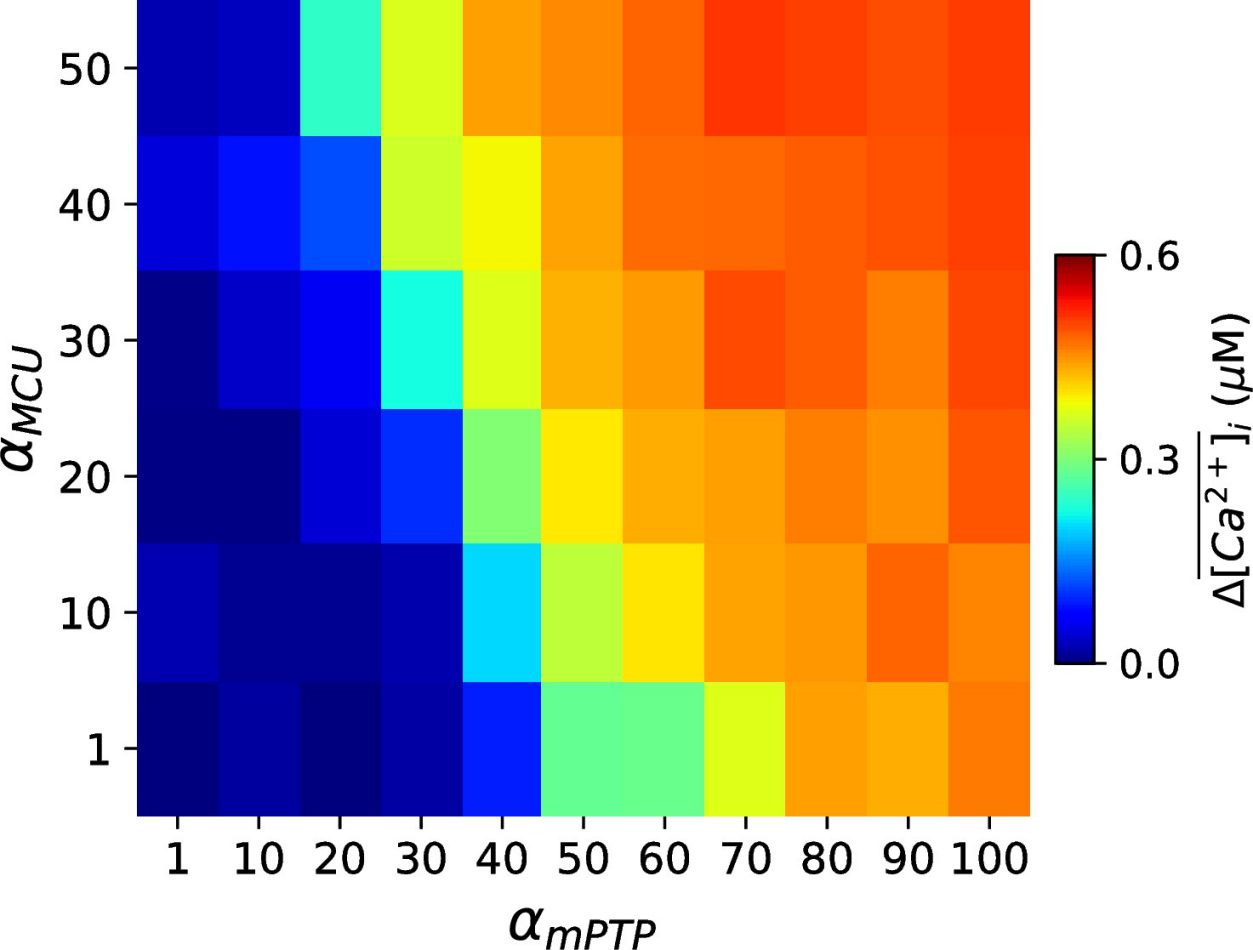
MCU upregulation promotes Ca^2+^ alternans through Ca^2+^- dependent opening of mPTP. Dependence of Ca^2+^ alternan amplitude $\Delta {\bar{\left[Ca^{2+}\right]}}_{i}$ on *α*_*MCU*_ and *α*_*mPTP*_. $\Delta {\bar{\left[Ca^{2+}\right]}}_{i}$ is calculated as the difference between the last two Ca^2+^ transient peaks in a simulation of 30 sec. PCL = 500 ms.

Under certain pathological conditions, such as nonischemic cardiomyopathy, MCU has been reported to dramatically upregulated [17], which may markedly increase the mitochondrial free Ca^2+^. Although our previous simulation study [40] has shown that the release of mitochondrial Ca^2+^ to the cytosol may only transiently affect the cytosolic Ca^2+^, the elevation of mitochondrial free Ca^2+^ is believed to promote the opening of mPTP and thus the production of ROS. Here we performed simulations to investigate how the MCU activity affects the genesis of Ca^2+^ alternans. For simplicity, we multiplied a pre-factor, *α*_*MCU*_, to the maximal MCU conductance. Therefore, *α*_*MCU*_ = 1 represents the control MCU conductance and increasing *α*_*MCU*_ increases the maximal MCU activity. Fig 3 shows the dependence of Ca^2+^ alternans amplitude on *α*_*MCU*_ and *α*_*mPTP*_. When the close-to-open rate of mPTP is low (*α*_*mPTP*_<20), no Ca^2+^ alternans occurs even if the maximal MCU activity is increased to 50-fold (*α*_*MCU*_ = 50). However, as mPTP open probability increases (i.e., as *α*_*mPTP*_ increases), the value of *α*_*MCU*_ required to generate Ca^2+^ alternans decreases. Furthermore, when the close-to-open rate of mPTP further increases (*α*_*mPTP*_>80), the effect of *α*_*MCU*_ on the genesis of Ca^2+^ alternans becomes less important. In addition, we found that inhibition of either MCU or mNCX in the setting of these simulations was unable to abolish Ca^2+^ alternans (S4 Fig). In conclusion, these results suggest that MCU upregulation may play an important role in generating Ca^2+^ alternans through mitochondrial Ca^2+^ mediated mPTP opening under certain pathological conditions.

### Effects of oxidized CaMKII signaling on Ca^2+^ alternans

**Fig 4:**
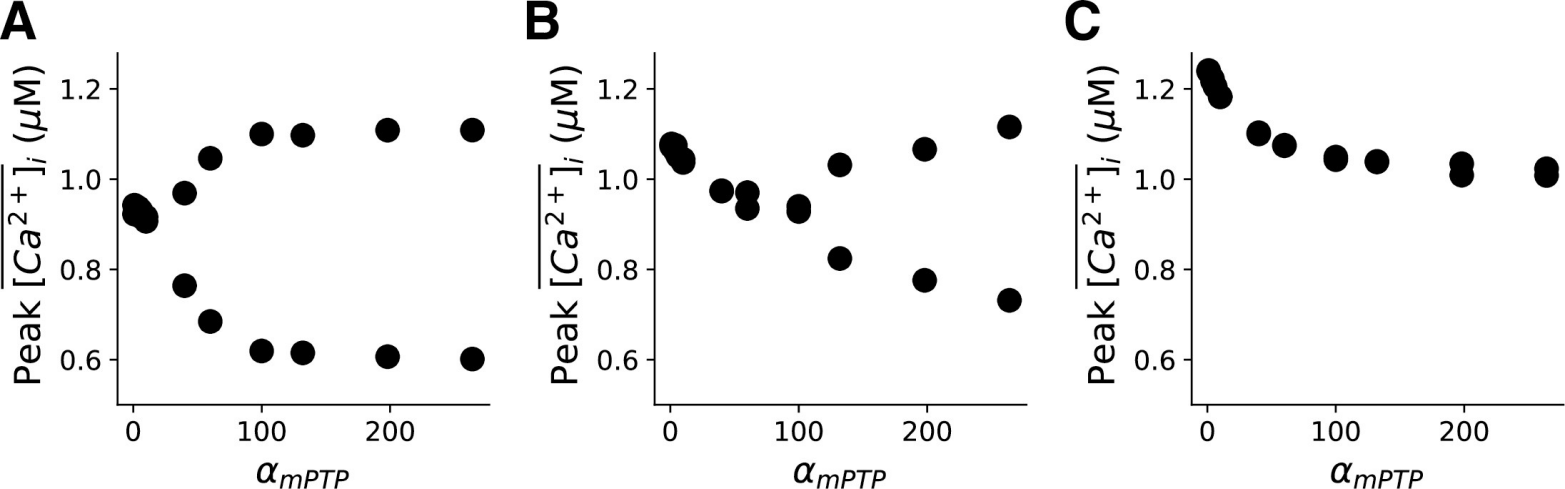
Effect of CaMKII activation on the genesis of the Ca^2+^ alternans. Bifurcation diagrams of peak values of Ca^2+^ transients in the last two beats vs. *α*_*mPTP*_ for clamped CaMKII activation level 1% (**A**), 10% (**B**) and 30% (**C**), respectively. PCL = 500 ms.

Since mitochondrial depolarization activates oxidized CaMKII signaling via ROS, we then evaluate the importance of CaMKII activation in the genesis of Ca^2+^ alternans during mitochondrial depolarization. As discussed earlier, CaMKII activation dynamics is a very slow process in the model and thus it can be treated as a quasi-steady state variable. Therefore, we clamped the CaMKII activation at different levels and examined the corresponding effects on the genesis of Ca^2+^ alternans. Fig 4 shows the bifurcation diagrams of the peak values of cytosolic Ca^2+^ transient against *α*_*mPTP*_ for CaMKII activity clamped at 1%, 10%, and 30% levels. These results show that Ca^2+^ alternans is suppressed as the CaMKII activation level increases. However, as shown in Fig 1A, the CaMKII activation level is about 1% for both control and the high mPTP open probability conditions within the 30 sec total simulation time. Therefore, our simulation results suggest that for the acute effect of mitochondrial depolarization on the genesis of Ca^2+^ alternans, CaMKII may not exhibit a big effect. However, for the long-term effect, CaMKII may play a more significant role, suppressing Ca^2+^ alternans via its regulation of SERCA.

### Effects of ATP depletion on Ca^2+^ alternans due to mitochondrial depolarization

When a mitochondrion depolarizes, it stops producing ATP, and as more mitochondria depolarize in the cell due to the increased mPTP open probability, the cytosolic ATP decreases. Since ATP is required for the SERCA pump, a low ATP level could impair the SERCA activity to promote Ca^2+^ alternans. As shown in Fig 1A, in our model, the cytosolic ATP level decays very slowly during mitochondrial depolarization. Similar to what we did for CaMKII, we clamped the cytosolic ATP concentration to different values and examined the consequences of ATP depletion during mitochondrial depolarization on the genesis of Ca^2+^ alternans.

**Fig 5:**
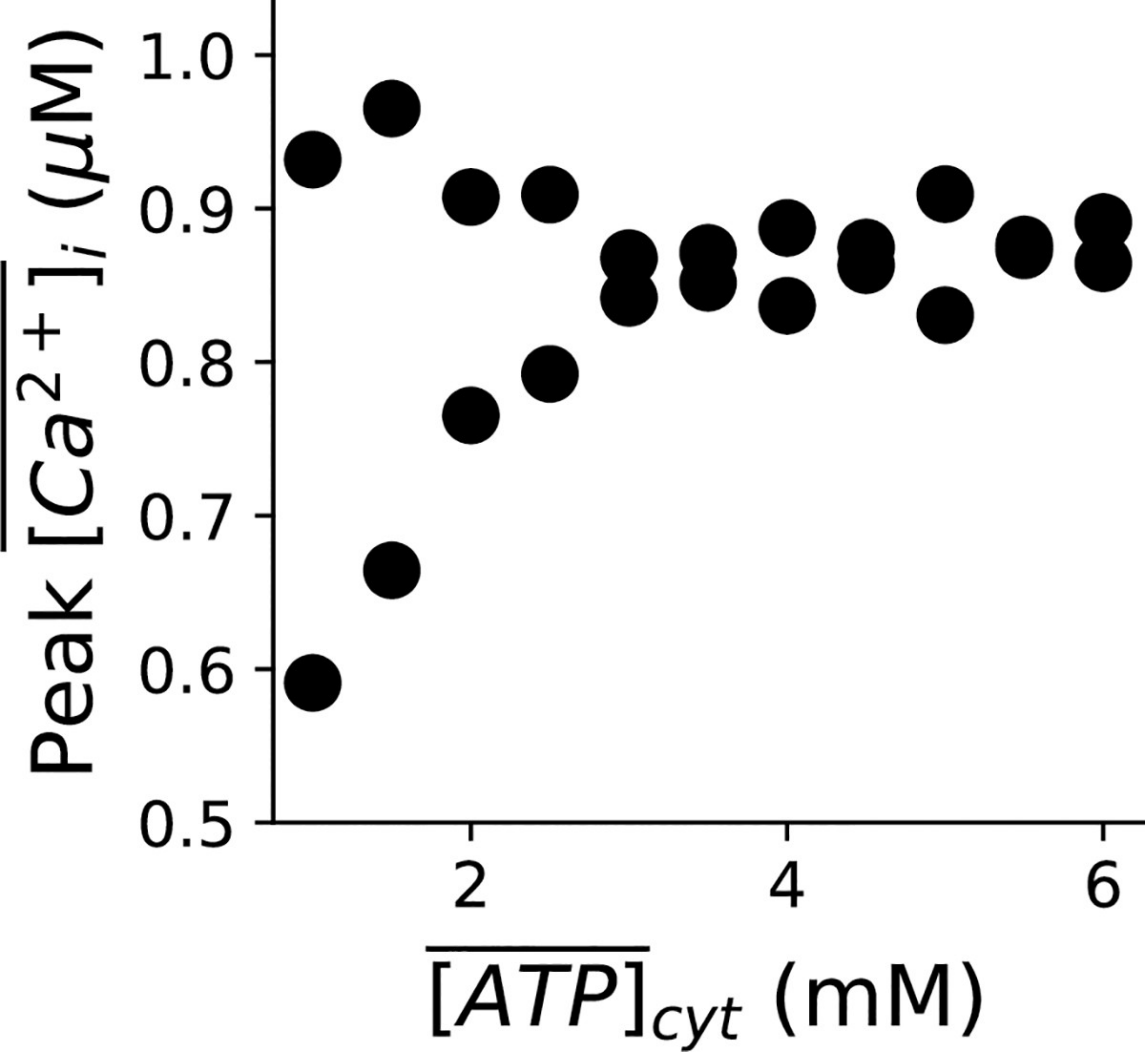
Effect of ATP on the genesis of Ca^2+^ alternans. Bifurcation diagrams of peak values of Ca^2+^ transients in the last two beats vs. clamped whole-cell average ATP level in the cytosol. *α*_*mPTP*_ = 30. The PCL = 500 ms.

Fig 5 shows the bifurcation diagram of the peak values of Ca^2+^ transient vs. the clamped cytosolic ATP concentration. The result shows that reducing the cytosolic ATP concentration promotes Ca^2+^ alternans during mitochondrial depolarization (*α*_*mPTP*_ = 30). This result suggests that the cytosolic ATP indeed has a great impact on the genesis of Ca^2+^ alternans. However, considering its slow decay during the process of mitochondrial depolarization, ATP may not play a central role in inducing Ca^2+^ alternans at least at the scale of sub-minute evolution. Therefore, similar to CaMKII, during mitochondrial depolarization, ATP may not exhibit a significant effect on the genesis of Ca^2+^ alternans acutely, but may promote alternans in a much longer time scale when the cytosolic ATP level reduces dramatically to impair the SERCA activity.

## Discussion

In this study, we used a physiologically-detailed ventricular myocyte model consisting of a 3D network of coupled CRUs and mitochondria to investigate the roles of mitochondrial depolarization via mPTP opening in the genesis of Ca^2+^ alternans. We showed that the effects of mitochondrial depolarization on Ca^2+^ alternans mainly via the ROS induced redox regulation of SERCA and RyRs. ROS induced oxidized CaMKII signaling and ATP decay exhibit little effect acutely but can exhibit large effects chronically via their regulations of SERCA. Mitochondrial Ca^2+^ cycling alone exhibits little effect on Ca^2+^ alternans but can exhibit a large effect via the Ca^2+^-dependent opening of mPTP.

### Role of ROS in the genesis of Ca^2+^ alternans

We have shown that an increase in the cytosolic ROS concentration in response to mitochondrial depolarization plays a key role in generating Ca^2+^ alternans. By judicious manipulation of the redox effects of ROS on RyRs and SERCA, we have been able to demonstrate that there is a synergy effect of the ROS redox regulation on both SERCA and RyRs in generating Ca^2+^ alternans. Our results show that only enhancing the RyR activity (Fig 2B, green) or reducing SERCA pump ability (Fig 2B, blue) via the ROS redox regulation causes a much smaller amplitude of Ca^2+^ alternans compared to the case where the ROS redox regulation effect is turned on for both RyRs and SERCA (Fig 2A, black). A previous study by Belevych et al. [23] has shown that an increase in ROS increases the RyR open probability, producing leaky RyR channels, and in turn resulting in Ca^2+^ alternans. Our findings here extend those of Belevych et al. [23], suggesting that the ROS redox regulation on SERCA is also crucial for promoting Ca^2+^ alternans. In fact, these findings well agree with the unified alternans theory developed in our previous study [42], since either hyperactivating RyRs or reducing the SERCA pump ability contributes to the increased steepness of the SR release-load relationship in cardiac myocytes, and therefore it is not surprising that the combined effect of the two factors synergistically promotes Ca^2+^ alternans.

Besides the redox effect, ROS is known to activate CaMKII, PKA and PKC pathways [30,31], which could affect the genesis of Ca^2+^ alternans. The model used in this study takes into account the effect of ROS on the activation of CaMKII by incorporating the oxidized CaMKII signaling formulation developed by Foteinou et al. [37]. The effect of CaMKII activation on Ca^2+^ handling proteins and ion channels has been simulated here following Hund and Rudy [36]. However, the kinetics of CaMKII appears to be very slow as seen in Fig 1A, since the percentage of CaMKII activation merely changes from 0.4% to 1.2%, whereas the mPTP open probability increases from ~0% to ~32% in response to the mitochondrial depolarization. These results therefore indicate that in the case of acute and severe metabolic insults, CaMKII activation may not be the cause of the genesis of Ca^2+^ alternans. In fact, our simulations of clamping CaMKII activation to different levels even suggest that higher CaMKII activation tends to suppress Ca^2+^ alternans (Fig 4) due to CaMKII phosphorylation of phospholamban, which reduces the half-maximal value of SERCA to increase SERCA pump activity. This agrees with our previous theoretical prediction that increasing SERCA pump activity can move the cell system out of the alternans regime [42]. However, that does not mean oxidized CaMKII activation during mitochondrial depolarization is beneficial, since CaMKII activation enhances LCC, RyRs and SERCA, which are known factors promoting arrhythmogenic Ca^2+^ waves, DADs and EADs [43–45].

Clinically, ischemia/reperfusion injury of myocardium is associated with both mitochondrial depolarization and repolarization phases. During the repolarization phase, Ca^2+^ homeostasis is disrupted, which is believed via opening of mPTP because of high ROS and mitochondrial Ca^2+^ accumulation [46–48]. However, some experiment showed that sustained depolarization of mitochondrial membrane potential did not occur even after 10 min of reperfusion [48], which requires more comprehensive future studies to explain. Nevertheless, our simulations suggest that ROS is the main factor for mitochondrial depolarization induced Ca^2+^ alternans, and targeting mPTP or ROS may prevent Ca^2+^ alternans in acute myocardial ischemia.

### Mitochondrial Ca^2+^ cycling and intracellular Ca^2+^ alternans

We have shown that MCU upregulation combined with the increased C_1_-to-O transition rate of mPTP gating kinetics promotes Ca^2+^ alternans (Fig 3). In our previous study [40], we found that mitochondrial Ca^2+^ released into the cytosol due to severe mitochondrial depolarization (100%) can only transiently change the cytosolic Ca^2+^ dynamics, but not the steady state. Therefore, our findings here indicate that mitochondrial Ca^2+^ may not directly affect the intracellular Ca^2+^ homeostasis, but it can enhance mPTP opening, which in turn causes Ca^2+^ alternans via ROS induced signaling pathways or lowering ATP. However, in nonischemic cardiomyopathy, MCU upregulation, together with other electrophysiological remodeling changes in heart failure conditions, can result in an all-or-none behavior or bistability, corresponding to the no EAD and EAD states in AP without mitochondrial depolarization [17]. Furthermore, the Ca^2+^ alternans in our simulations is driven by the cytosolic Ca^2+^, not the mitochondrial Ca^2+^. As shown in S5 Fig, when the mitochondrial Ca^2+^ was clamped to fixed values, the cytosolic Ca^2+^ alternans still exists (S5A Fig), but when cytosolic Ca^2+^ was clamped to fixed values, the mitochondrial Ca^2+^ alternans disappeared (S5B Fig).

### ATP level and Ca^2+^ alternans

Previous experimental studies have documented that a reduced cellular ATP level is linked to Ca^2+^ alternans[14,24,25], presumably via the reduced SERCA pump activity. Here, we have shown that the ATP level during acute mitochondrial depolarization (30%) only changes slightly in our simulation (Fig 1A). If that is the case in experiment, then it is unlikely that the Ca^2+^ alternans occurring during acute mitochondrial depolarization is associated with the cellular ATP change. However, in experiment, whether the cellular ATP level is reduced has been shown to depend on the concentration of FCCP and treatment time [49]. Because the newly developed experimental technology can now be used to measure the ATP level in living functioning cells [50,51], future work should therefore include the fine-tuning of the ATP computer model to match the dynamics of ATP in individual experiments. Here, in order to evaluate the role of the ATP level in the genesis of Ca^2+^ alternans, we have performed simulations to clamp the cytosolic ATP concentration at different levels during mitochondrial depolarization. We show that when ATP is low enough, it can promote alternans via its effect on SERCA. However, it could work in synergy with the redox effect of ROS on SERCA even at much higher levels. Note that the effect of ATP on the genesis of Ca^2+^ alternans under mitochondrial depolarization is also mPTP dependent, since different mPTP open probabilities would cause different levels of cytosolic ROS, thereby affecting the cytosolic Ca^2+^ dynamical regime, which is on top of the ATP effect on Ca^2+^ alternans (S6 Fig). Also, the cytosolic ATP concentration itself depends on the mPTP activity; the opening of mPTP depolarizes the mitochondrial membrane potential, malfunctioning the ATP synthases.

### Limitations

Several limitations should be noted in this study. The AP model and the 3-dimensional CRU network used in this study can successfully simulate the basic excitation-contraction-metabolism coupling in a ventricular myocyte, but it cannot capture all the aspects of electrophysiology of a real myocyte, such as heterogeneities in T-tubule networks and distribution of ion channels and Ca^2+^ handling proteins [52,53]. Such heterogeneities may alter the propensity of a cardiac myocyte for alternans [9]. Moreover, in this study the opening of mPTP was only mitochondrial free Ca^2+^ dependent, and the open probability was increased by increasing the C_1_ to O transition rate constant. However, mPTP could open via a ROS-induced ROS release mechanism [54,55]. This mechanism is important for modeling mitochondrial depolarization waves [56–58], which are not the focus of this study. Furthermore, mitochondria have been found to constantly divide and fuse in cardiac myocytes, and in heart failure conditions, mitochondrial fusion could be depressed, which may contribute to the genesis of cardiac arrhythmias [59]. Therefore, further advanced computer models should be developed in the future to incorporate the feature of mitochondrial fusion and fission. Lastly, mitochondria have a special BK channels (mBKs), which is a voltage-dependent and Ca^2+^-activated K^+^ channel with a conductance ~100–300 pS. It has been known that the opening of mBK brings in K^+^ into the mitochondrion, depolarizing Δψ. It then reduces the driving force of MCU, which in turn attenuates the overload of mitochondrial Ca^2+^ (Stowe et al. [60]). The cardioprotective role of the mBK channel has been proposed to be similar to the mKATP channel (Stowe et al. [60]). The opening of these channels may prevent mPTP from opening by reducing mitochondrial Ca^2+^ overload. Therefore, in future studies, models of mBK and mKATP channels will be developed and incorporated in the mitochondrion model.

## Methods

The details of this model, including mathematical formulation, values of parameters, and experimental validation, can be found in Song et al. [40]. Here we describe some of the key components of the model that are important for this study.

### The overall ventricular myocyte model structure

This rabbit ventricular myocyte model contains a 3-dimensional coupled network of CRUs and mitochondria. There are 21504 (64×28×12) CRUs and 5376 (64×14×6) mitochondria (see Song et al. [40] for the details on the arrangement of these networks.).The membrane potential (V) of the cell is described by

$$C_{m}\frac{dV}{dt}=I_{Na}+I_{Na,L}+I_{Ca,L}+I_{NCX}+I_{K1}+I_{Kr}+I_{Ks}+I_{to,f}+I_{to,s}+I_{NaK}+I_{K,ATP}+I_{Ca,b}-I_{sti}$$ (1)

where *C*_*m*_ = 1 μF/cm^2^ is cell membrane capacitance, and *I*_*sti*_ is the stimulus pulse with the current density being -80 μA/cm^2^ and the duration being 0.5 ms. The formulations of the ionic currents are referred to Song et al. [40].

The Gilespie method was used to simulate the random transitions of LCCs, RyRs, and mPTPs. The Euler method was used to solve the differential equations, and an adaptive time step method was used to compute the AP upstroke [61] with a time step 0.001 ms. The time step for computation for the rest of the AP was 0.01 ms. The computer model was programmed in CUDA C++ with double precision on Nvidia Tesla K20c and K80 GPU cards.

### ROS and CaMKII regulation of RyRs

Both oxidized CaMKII signaling and the redox regulation of ROS increase the RyRs open probability [30,31,62–64]. To model these effects, we formulated the close-to-open rate (*k*_12_) of RyRs as follows:

$$k_{12}=k_{base}k_{u}(1+\Delta k_{CaMKII}+\Delta k_{ROS}){\left({\left[Ca^{2+}\right]}_{p}\right)}^{2}$$ (2)

where Δ*k*_*CaMKII*_ and Δ*k*_*ROS*_ are the CaMKII-dependent and ROS-dependent components, respectively. The equations of Δ*k*_*CaMKII*_ and Δ*k*_*ROS*_ are formulated in Song et al. [40].

$$\Delta k_{CaMKII}=\frac{\Delta k_{CaMK,max}}{1+{\left(\frac{K_{mCaMRyR}}{{\left[CaMKII\right]}_{act}}\right)}^{h_{CaMRyR}}}$$ (3)

$$\Delta k_{ROS}=\frac{\Delta k_{ROS,max}}{1+{\left(\frac{K_{mROSRyR}}{{\left[ROS\right]}_{cyt}}\right)}^{h_{ROSRyR}}}$$ (4)

In Fig 2B, when we examined the effect of ROS via SERCA alone on inducing Ca^2+^ alternans, we set Δ*k*_*ROS*_ = 0 to remove the effect of ROS on RyR activity. Other than that, Δ*k*_*ROS*_ was calculated using Eq 4. *k*_*base*_ and *k*_*u*_ are rate constants. [*Ca*^2+^]_*p*_ is the Ca^2+^ concentration in the dyadic space. *k*_12_ represents a closed-to-open rate of the RyR model [40] incorporated in the cell model. Increasing *k*_12_ increases the open probability of RyRs.

### ROS and CaMKII regulation of SERCA pump

Direct redox regulation slows the SERCA pump activity [31], and CaMKII phosphorylation of the phospholamban reduces the half maximum value [36]. Hence, the CaMKII effect on the activity of SERCA competes with the ROS effect on the SERCA activity. SERCA activity is also influenced by the ATP [65], i.e., reducing ATP impairs the SERCA pump function. Thus, the formulation of SERCA activity incorporating the ATP, CaMKII and ROS dependency is as follows:

$$J_{up}{=v}_{up}f_{up,ATP}f_{up,ROS}\frac{{\left[Ca^{2+}\right]}_{i}^{2}}{{\left[Ca^{2+}\right]}_{i}^{2}+{\left(K_{i}-PLB({\left[CaMKII\right]}_{act})\right)}^{2}}$$ (5)

where *f*_*up*,*ATP*_, and *f*_*up*,*ROS*_ are ATP and ROS-dependent functions, which are detailed in Song et al. [40].

$$f_{up,ATP}=\frac{1}{1+\frac{{\left[ADP\right]}_{f}}{k_{i,up}^{\prime }}+(1+\frac{{\left[ADP\right]}_{f}}{k_{i,up}})\frac{k_{mupATP}}{\left[ATP\right]}}$$ (6)

$$f_{up,ROS}=\frac{1}{1+{\left(\frac{{\left[ROS\right]}_{cyt}}{k_{d,ROS}}\right)}^{h_{ROS,SERCA}}}+\frac{0.75}{1+{\left(\frac{k_{d,ROS}}{{\left[ROS\right]}_{cyt}}\right)}^{h_{ROS,SERCA}}}$$ (7)

Eq 6 was adopted from Cortassa et al. [65]. Eq 7 was formulated in our previous study [40] to account for the redox effect of ROS on SERCA. In Fig 2B, when we examined the effect of ROS via RyR on inducing the Ca^2+^ alternans, we set *f*_*up*,*ROS*_ = 1 to eliminate the redox effect of ROS on SERCA in our model. Other than that, *f*_*up*,*ROS*_ was calculated using Eq 7 in this study. *v*_*up*_ and *k*_*i*_ is the maximum SERCA strength and the half maximum value, respectively. *PLB*([*CaMKII*]_*act*_) is a CaMKII dependent function affecting the half maximum value of SERCA, where [*CaMKII*]_*act*_ is the local CaMKII activation in the cytosol.

### The mPTP model

A 3-state (two close states C_0_ and C_1_, and an open state O) model of the mPTP (S7 Fig) was used. The transition from the C_0_ state to the C_1_ state is mitochondrial free Ca^2+^ dependent as shown below:

$$k_{c0c1}=\alpha_{0}\left(1+199\times \frac{{\left[{Ca}^{2+}\right]}_{m}^{h_{mPTP}}}{{\left[{Ca}^{2+}\right]}_{m}^{h_{mPTP}}+{\left[{Ca}^{2+}\right]}_{0}^{h_{mPTP}}}\right)$$ (8)

Where *h*_*mPTP*_ is the Hill coefficient, [Ca^2+^]_m_ is the mitochondrial free Ca^**2+**^ in the corresponding mitochondrion, and [Ca^**2+**^]_**0**_ is the half maximum value. Other transition rates are assumed to be constant. To simulate different levels of mPTP open probability, we multiplied a pre-factor, *α*_*mPTP*_, to the C_1_ to O transition rate, $k_{c1o}^{0}$,

$$k_{c1o}=\alpha_{mPTP}\cdot k_{c1o}^{0}$$ (9)

The relationship between *α*_*mPTP*_ and the steady state open probability of mPTP is the following,

$$P_{mPTP}=\frac{1}{1+\frac{1}{\alpha_{mPTP}(\frac{k_{oc1}}{k_{c1o}^{0}}+\frac{k_{c1c0}}{k_{c0c1}}\cdot \frac{k_{oc1}}{k_{c1o}})}}$$ (10)

Eq 10 suggests that *P*_*mPTP*_~0 when *α*_*mPTP*_~0, and *P*_*mPTP*_~1 when *α*_*mPTP*_~ ∞. Therefore, by simply increasing *α*_*mPTP*_, we were able to increase the level of the open probability of mPTP.

## Supporting information

S1 Fig From top to bottom, time traces of mitochondrial membrane potential, whole-cell averaged cytosolic ROS level, whole-cell averaged cytosolic Ca^2+^ concentration. The red bar indicates the time period when all the mPTPs in the cell were commanded to be closed.
(TIF)

S2 Fig **A.** Time traces of voltage and Ca for control and mitochondrial depolarization cases at PCL = 500 ms. **B.** Time traces of voltage and Ca for mitochondrial depolarization case at PCL = 300 ms. Note that we plot two consecutive beats on top of each other to better observe the alternans.
(TIF)

S3 Fig Bifurcation diagram of peak values of Ca^2+^ transients vs. the whole-cell averaged open probability of mPTP. The open probability of mPTP was measured at the end of each simulation of Fig 2A. We can see that the threshold for mPTP open probability is ~20% in this model. However, this specific value of the threshold depends on many factors, such as what we discussed in the Limitation section.
(TIF)

S4 Fig **A.** Time traces of whole-cell averaged cytosolic Ca^2+^ transient and mitochondrial Ca^2+^ for *α*_*MCU*_ = 1 (red) and 0.5 (black). *α*_*mPTP*_ = 60. **B.** Same as A, but mitochondrial NCX was at control (red) and 50% reduction (black). *α*_*mPTP*_ = 60, PCL = 500 ms.
(TIF)

S5 Fig **A.** Time traces of the whole-cell average cytosolic Ca^2+^, mitochondrial Ca^2+^, where the mitochondrial Ca^2+^ was clamped to 0.4 (black), 0.5 (red), and 0.6 (blue) μM at t = 25 sec. **B.** Same as A, but the cytosolic Ca^2+^ was clamped to 0.2 (black), 0.5 (red), and 1 (blue) μM at t = 25 sec.
(TIF)

S6 FigDependence of Ca^2+^ alternans amplitude on *α*_*mPTP*_ and ${\bar{\left[ATP\right]}}_{cyt}.\Delta {\bar{\left[Ca^{2+}\right]}}_{i}$ was calculated as the difference between the last two Ca^2+^ transient peaks in a simulation of 30 sec. PCL = 500 ms.(TIF)

S7 Fig Three-state mPTP model. C_0_ and C_1_ are the two closed states. O represents the open state.
(TIF)
