# Supplementary figures and images for: Mitochondrial depolarization promotes calcium alternans: Mechanistic insights from a ventricular myocyte model
Source: PLoS Comput Biol. 2021 Jan 25;17(1):e1008624. doi: 10.1371/journal.pcbi.1008624 (PMC7861552; doi:10.1371/journal.pcbi.1008624)

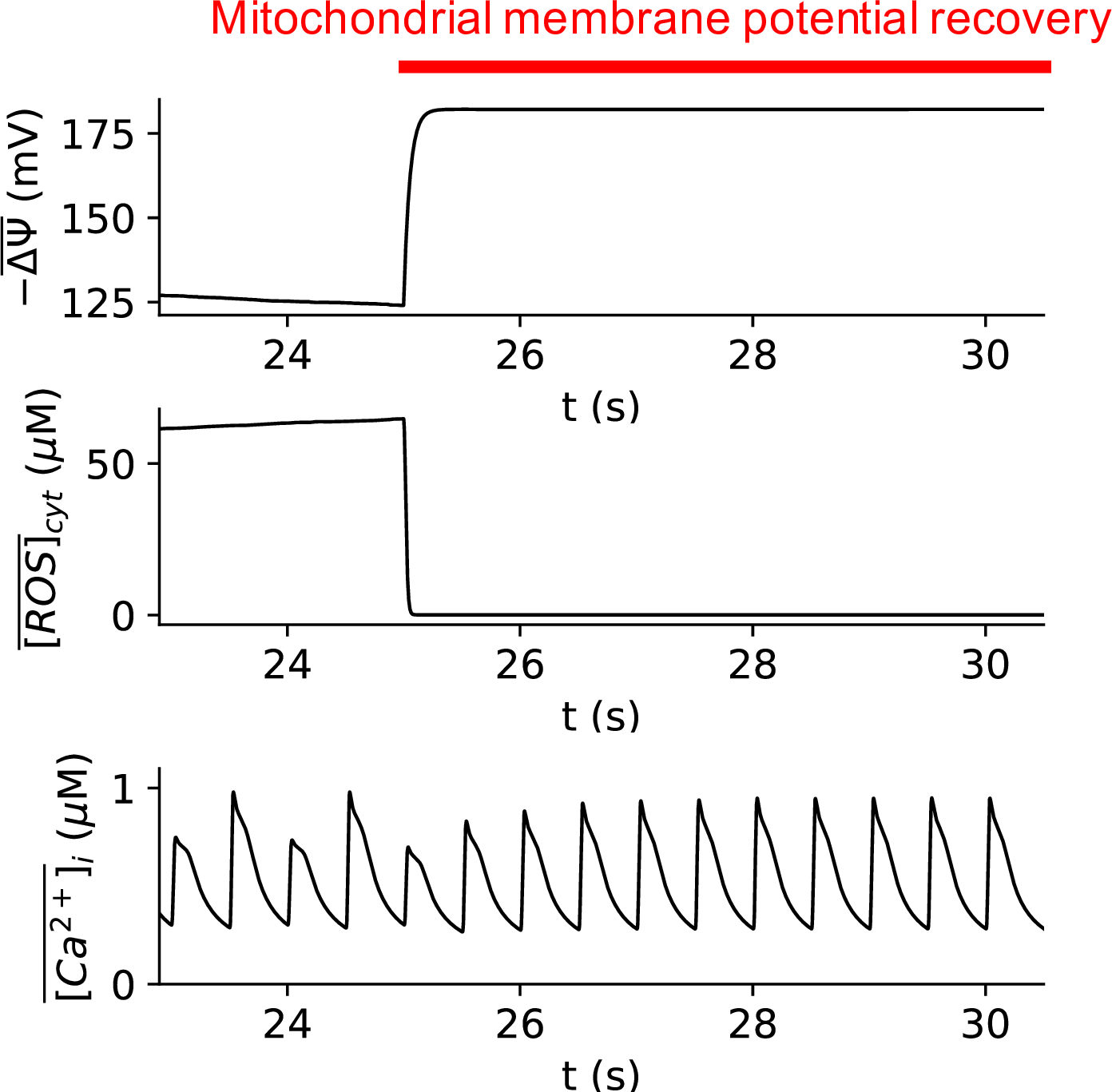

Supplement: S1 Fig — From top to bottom, time traces of mitochondrial membrane potential, whole-cell averaged cytosolic ROS level, whole-cell averaged cytosolic Ca2+ concentration. The red bar indicates the time period when all the mPTPs in the cell were commanded to be closed. (TIF) [file pcbi.1008624.s001.tif]

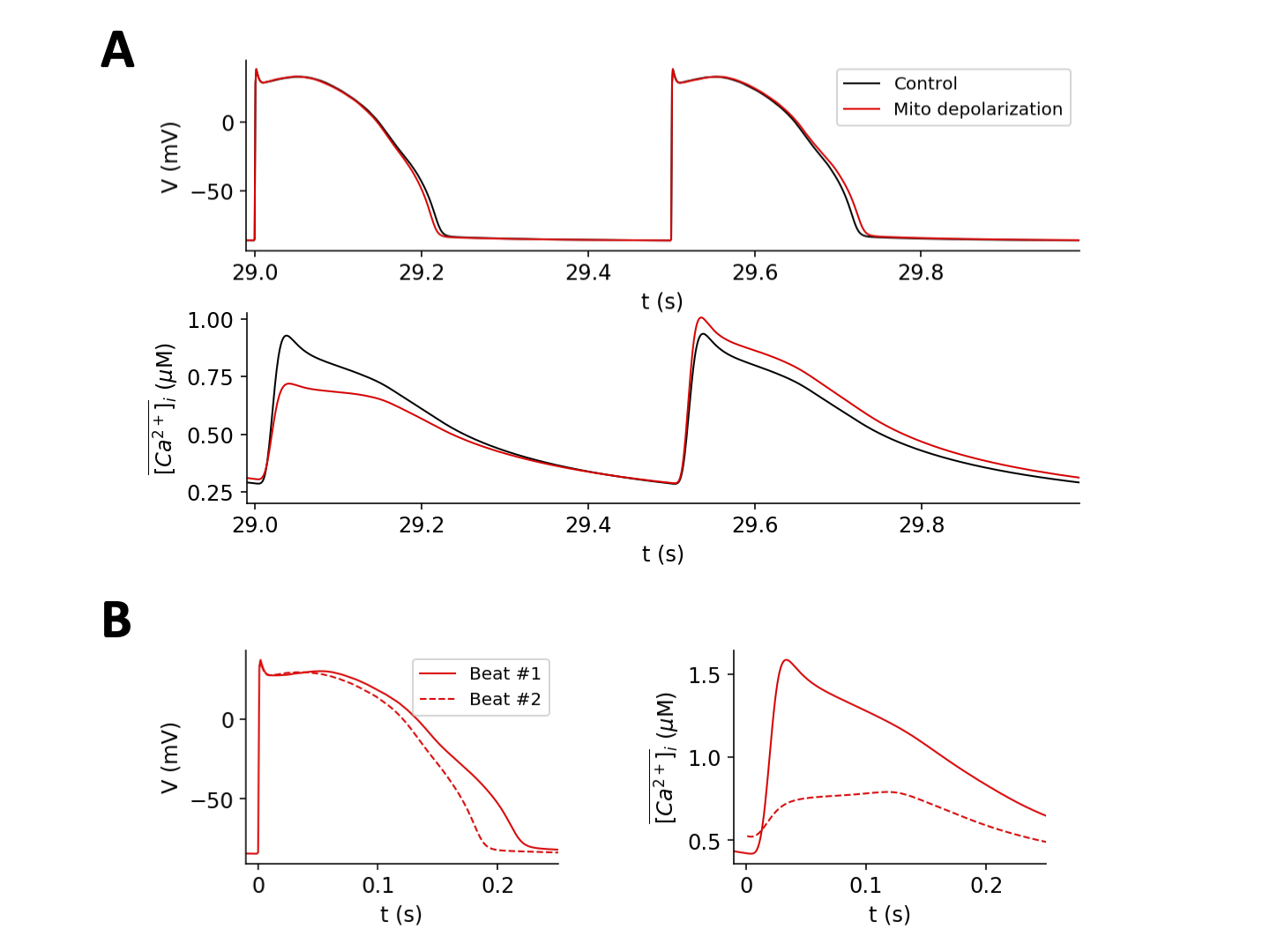

Supplement: S2 Fig — A. Time traces of voltage and Ca for control and mitochondrial depolarization cases at PCL = 500 ms. B. Time traces of voltage and Ca for mitochondrial depolarization case at PCL = 300 ms. Note that we plot two consecutive beats on top of each other to better observe the alternans. (TIF) [file pcbi.1008624.s002.tif]

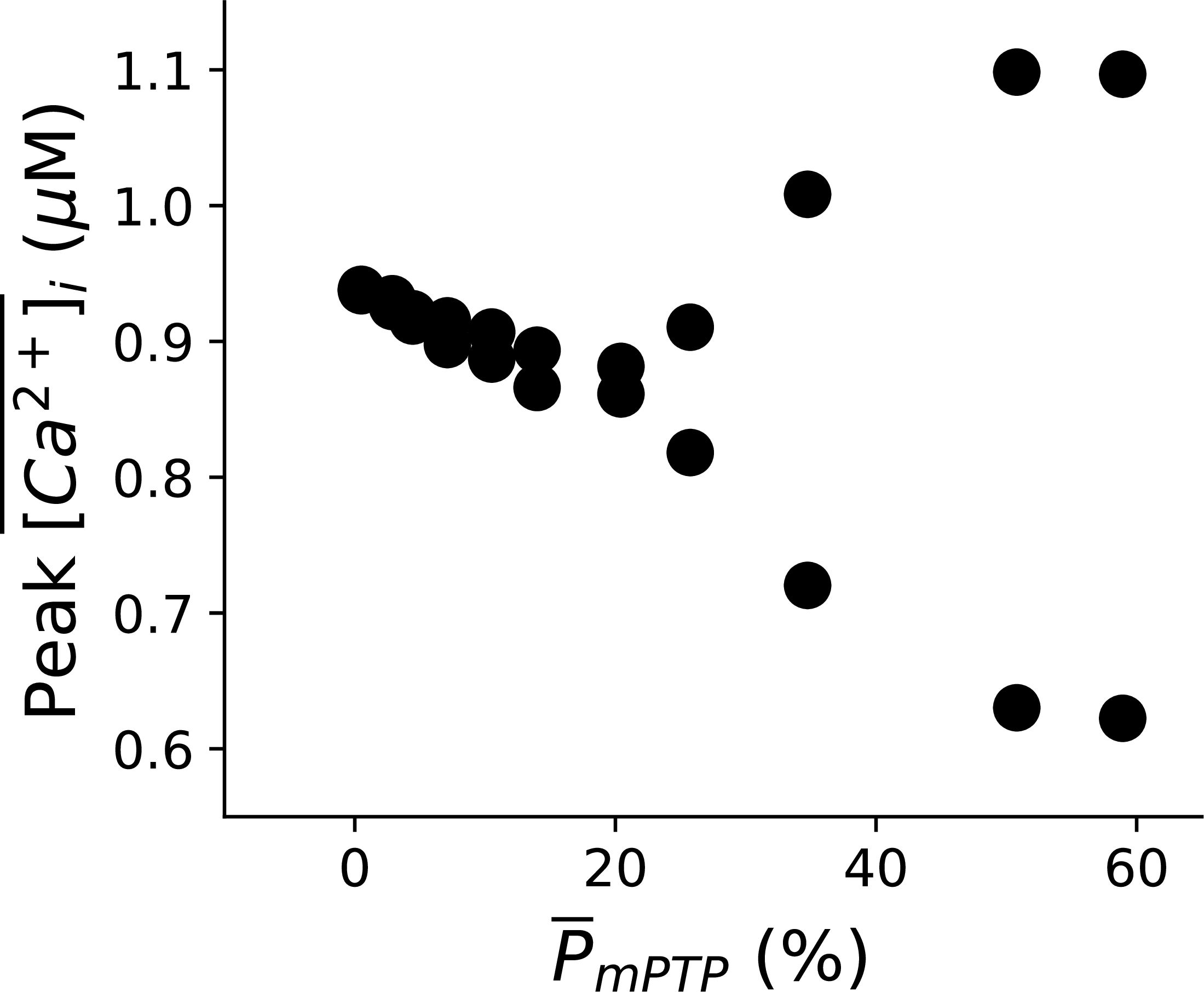

Supplement: S3 Fig — Bifurcation diagram of peak values of Ca2+ transients vs. the whole-cell averaged open probability of mPTP. The open probability of mPTP was measured at the end of each simulation of Fig 2A. We can see that the threshold for mPTP open probability is ~20% in this model. However, this specific value of the threshold depends on many factors, such as what we discussed in the Limitation section. (TIF) [file pcbi.1008624.s003.tif]

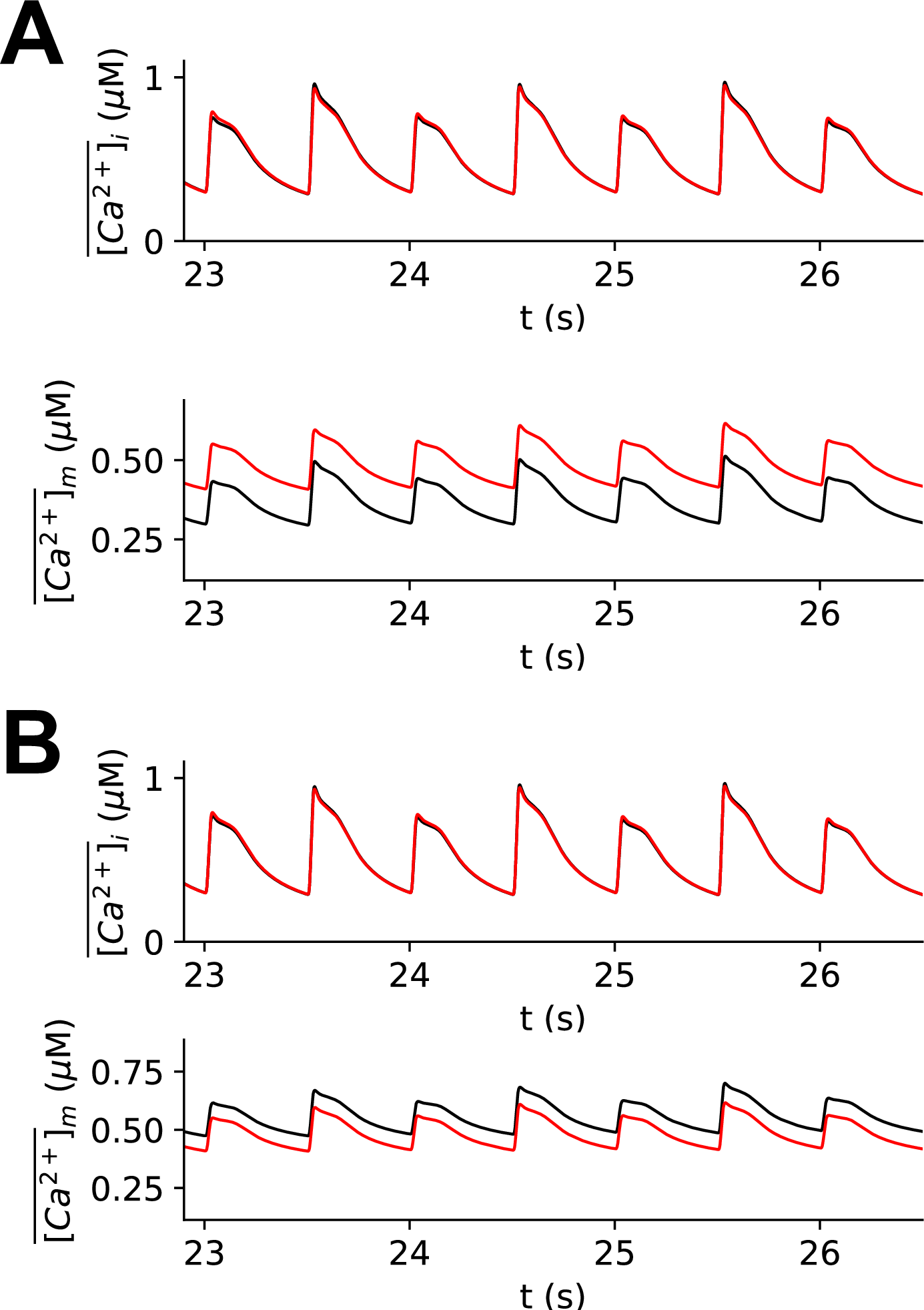

Supplement: S4 Fig — A. Time traces of whole-cell averaged cytosolic Ca2+ transient and mitochondrial Ca2+ for αMCU = 1 (red) and 0.5 (black). αmPTP = 60. B. Same as A, but mitochondrial NCX was at control (red) and 50% reduction (black). αmPTP = 60, PCL = 500 ms. (TIF) [file pcbi.1008624.s004.tif]

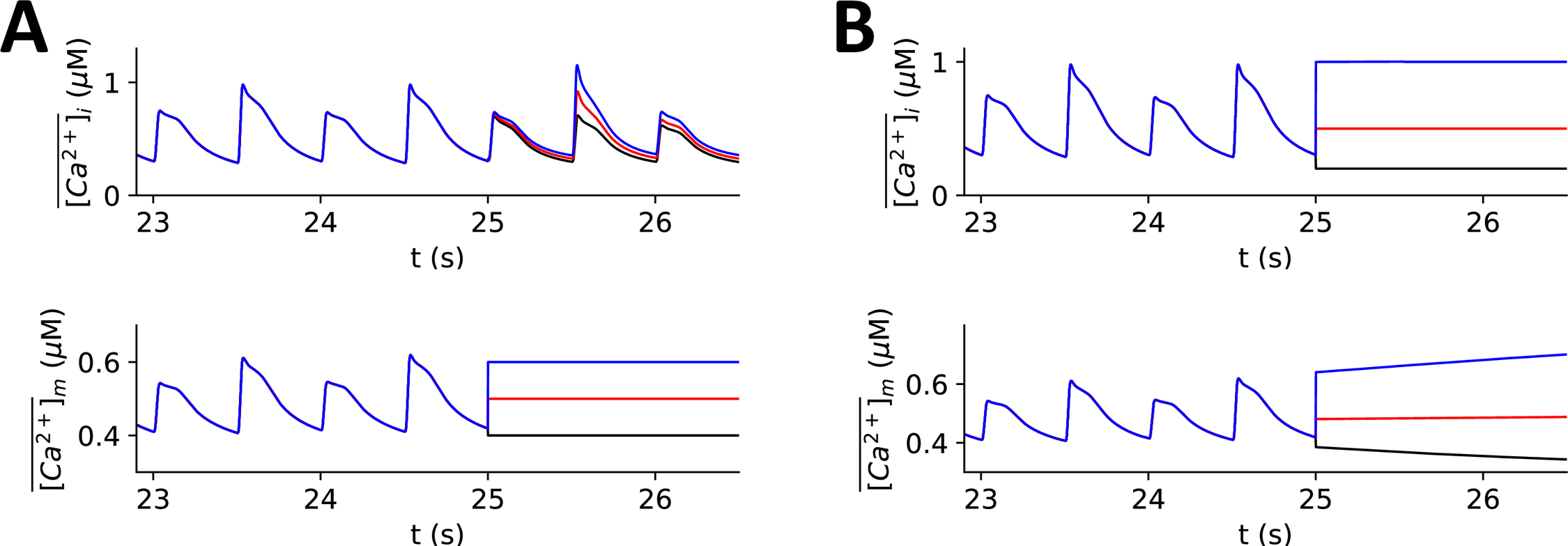

Supplement: S5 Fig — A. Time traces of the whole-cell average cytosolic Ca2+, mitochondrial Ca2+, where the mitochondrial Ca2+ was clamped to 0.4 (black), 0.5 (red), and 0.6 (blue) μM at t = 25 sec. B. Same as A, but the cytosolic Ca2+ was clamped to 0.2 (black), 0.5 (red), and 1 (blue) μM at t = 25 sec. (TIF) [file pcbi.1008624.s005.tif]

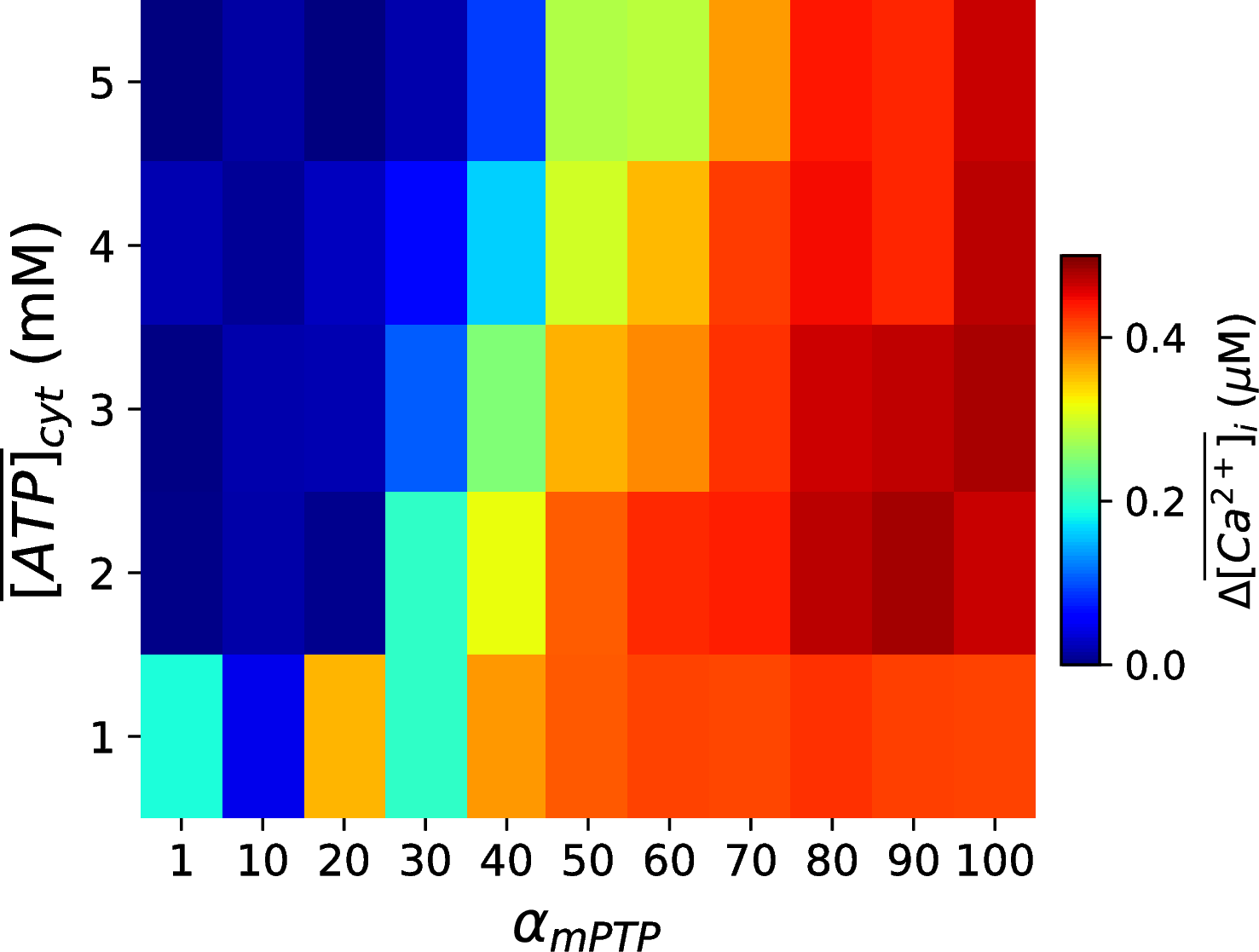

Supplement: S6 Fig — Dependence of Ca2+ alternans amplitude on αmPTP and [ATP]¯cyt.Δ[Ca2+]¯i was calculated as the difference between the last two Ca2+ transient peaks in a simulation of 30 sec. PCL = 500 ms. (TIF) [file pcbi.1008624.s006.tif]

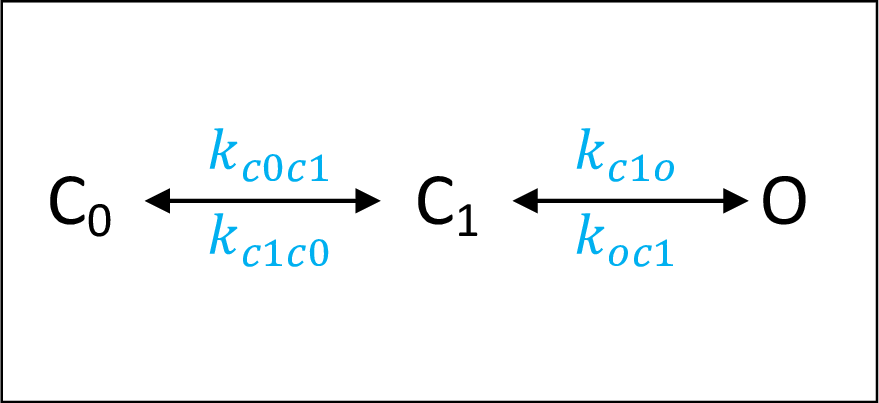

Supplement: S7 Fig — Three-state mPTP model. C0 and C1 are the two closed states. O represents the open state. (TIF) [file pcbi.1008624.s007.tif]
